# Gangrenous Metastasis from the Lungs to the Brain

**Published:** 1853-05

**Authors:** Rud. Virchow


*Gangrenous Metastasis from the Lungs to the Brain.* By Rud. Virchow.—I have already, in my work upon inflammation of arteries, (Virchow's Archiv., Bd. I., S. 332,) communicated a case where, in a patient who died of gangrene of the lungs, there were found in the mesentery gangrenous deposits, which more minute investigation proved to be lodged within the branches of the mesenteric artery, such deposits having come from the gangrenous lung by the pulmonary veins, and been propelled from the left side of the heart into the systemic circulation. As this form of metastasis has not yet been much investigated, the relation of a fresh case may serve to arouse the attention of pathologists.

Anna Maria Schwing, of Buchold, aged 21, came to the Julius Hospital, May 18th, 1852, suffering from melancholia religiosa, accompanied by occasional maniacal fits. From June 10th to two days before her death, she exhibited the most determined abhorrence of food. From July 8th, there were symptoms of severe affection of the lungs, characterised by fetid and blood-stained sputa, paroxysms of asthma, and uncontrollable fits of cough. Her excited state prevented a more accurate examination. Death ensued August 8th, at a quarter past seven A. M., without any further cerebral symptoms.

*Examination of the Body*, August 9.—Skull-cap thin, but normal; sinuses empty, but superficial; cerebral veins full; numerous granulations (gland. Pacchioni?) along the sinus long.; considerable serous effusion at the base of the skull and in the ventricles; cerebral substance firm. In the left ventricle, and extending over both the optic thalamus and corpus striatum, there was a discoloured, dirty grey surface, under which was a whitish semifluid, or crumbling mass of limited extent. Upon further examination, four similar spots were seen upon the posterior surface of the hemispheres, under the pia mater, and in the sulci; they consisted of a white, semifluid, stinking substance, surrounded, as in the ventricle, by a dirty grey circumference. The microscopical appearances of this substance showed that it consisted of an amorphous granular detritus, with long, spear-shaped, fat crystals, (such as I have shown to occur often in gangrene of the lung,) pigment masses, and altered blood-discs. Parts of the mass were traced, under the microscope, into the arteries of the pia mater.

Old adhesions on both sides of the chest; recent effusions of lymph over gangrenous portions of the visceral pleura. The right lung was torn in separating its adhesions; and, from the fissure, there issued a thick, dirty, grey, and partially brownish, stinking substance, which had been enclosed in a cavity with defined walls. There were many other similar accumulations scattered throughout the pulmonary substance, one as large as a hen's egg. The microscopic characters corresponded with those mentioned in the cerebral deposit.

It may be inferred, that, in consequence of imperfect nutrition, gangrene of the lung first ensued; then small granular particles became detached, entered the left side of the heart by the pulmonary veins, and thence were driven through the carotids to the pia mater, where they spread the decomposing process to the structure of the brain. The spots were of limited extent, the surrounding vessels and tissues being normal.—*Virchow's Archiv.*, 1853.

Dr. Kirkes observes, in speaking of the detachment of fibrinous deposits from the interior of the heart: "The finely granular material resulting from their disintegration, mingling and circulating with the blood, may give rise to various disturbances indicative of a contaminated state of this fluid, producing symptoms very similar to those observed in phlebitis, typhus, and other analogous diseases." This remark may be extended to disorganization going on in the lungs, and perhaps in other organs; and the cases related by the different authors who have written upon this subject should stimulate those upon whom the responsibility of making *post-mortem* examinations falls, to investigate, more thoroughly than has heretofore been done, the condition of the interior of arteries and veins, both in their main trunks and in the immediate proximity of diseased tissues.—*Lon. Med. Times & Gaz.*

